# Presence of localized elevated metabolic enzymes and *kdr* mutations in the voltage-gated sodium channel gene indicate early evidence of resistance of *Phlebotomus argentipes* to alpha-cypermethrin in Bihar, India

**DOI:** 10.1186/s13071-026-07339-8

**Published:** 2026-03-31

**Authors:** Ashish Shukla, Rahul Chaubey, Anurag Kumar Kushwaha, Urvashi Geeta Rai, Rajiv Kumar, Christine Petersen, Philip Lawyer, Edgar Rowton, Scott A. Bernhardt, Shyam Sundar

**Affiliations:** 1 Department of Medicine, Institute of Medical Sciences, Banaras Hindu University, Varanasi, India; 2 Kala-Azar Medical Research Centre, Muzaffarpur, Bihar India; 3 Centre of Experimental Medicine and Surgery, Institute of Medical Sciences, Banaras Hindu University, Varanasi, Uttar Pradesh India; 4 Department of Veterinary Biosciences, Ohio State University, Columbus, Ohio USA; 5 Arthropod Collections, Monte L. Bean Life Science Museum, Brigham Young University, Provo, Utah USA; 6 Division of Entomology, Walter Reed Army Institute of Research, Silver Spring, MD USA; 7 Department of Biology, Utah State University, Logan, Utah USA

**Keywords:** *Ph. argentipes*, Metabolic detoxification, *Vgsc* gene, *kdr* mutation, Bihar, India

## Abstract

**Background:**

Visceral leishmaniasis, caused by *Leishmania donovani* and transmitted by *Phlebotomus argentipes*, remains a major public health challenge in the Indian subcontinent. Sand fly populations are controlled by using different insecticides, particularly dichlorodiphenyltrichloroethane (DDT) and pyrethroids such as alpha-cypermethrin. Prolonged and irregular use of these insecticides raises concern about the development of resistance in sand fly populations.

**Methods:**

This study aimed to compare metabolic detoxification and knockdown resistance (*kdr*) mutations in *Vgsc* and *ace-1* between nine sprayed (indoor residual spray, IRS) villages and one unsprayed (non-IRS) village in Muzaffarpur, Bihar, India. A total of 10 *Ph. argentipes* from each village were used for metabolic detoxification and 10–25 *Ph. argentipes* were used for target site insensitivity mutations. Homogenized *Ph. argentipes* aliquots were used for different enzymes activity and DNA was used for the sequence analysis of *Vgsc* and *ace-1* genes to access the presence of *kdr* mutations.

**Results:**

Biochemical assays revealed that levels of detoxification enzymes, including glutathione S-transferase (GST), esterase (PNPA), and cytochrome P450 monooxygenase, were elevated in IRS villages, suggesting localized metabolic resistance. Molecular screening of the voltage-gated sodium channel (*Vgsc*) gene revealed high frequencies of knockdown resistance (*kdr*) mutations at codon 1014, with serine (L1014S) (48.5%) being the most prevalent, followed by wild type leucine (L1014) (39.5%). No mutations were detected at codon 119 of the *ace-1* gene, indicating the sensitivity to organophosphates in the sand fly population.

**Conclusions:**

The results suggested that continuous and repeated exposure to the synthetic pyrethroid may exert selective pressure, leading to early signs of resistance in *Ph. argentipes*, mediated through metabolic detoxification mechanisms and mutation in the *kdr* gene. These findings underscore the importance of ongoing resistance monitoring and the implementation of rotational insecticide strategies to support sustained efforts toward the elimination of visceral leishmaniasis.

**Graphical Abstract:**

**Figure:**
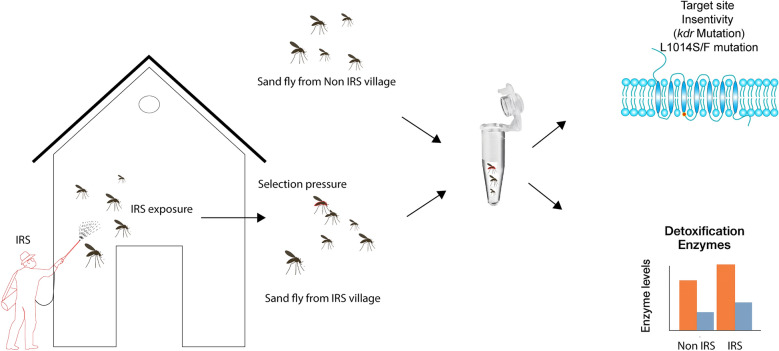

**Supplementary Information:**

The online version contains supplementary material available at 10.1186/s13071-026-07339-8.

## Background

Visceral leishmaniasis (VL) is a vector-borne disease transmitted by *Phlebotomus* sand flies in the Old World and *Lutzomyia* in the New World that can be lethal if not treated. On the Indian subcontinent (ISC), VL is caused by the obligatory intracellular protozoan *Leishmania donovani* [1, 2]. Early diagnosis and treatment of cases and integrated vector control are key efforts in the management of leishmaniasis. There has been an increase in the prevalence of VL in several countries where case management is the core of leishmaniasis control [3]. The VL elimination program depends on the application of synthetic insecticides for vector control. Initially, during the time between 1953 and 1962, when dichlorodiphenyltrichloroethane (DDT) was implemented as the principal modality of the National Malaria Eradication Program, there was an immense effect in reducing sand fly populations and a significant decline in the number of VL cases [4]. DDT was the only insecticide used by the Indian VL control program until 2015. Several studies revealed that sand flies had become resistant to DDT [5, 6]. As tolerance for DDT increased, the National Kala-Azar Elimination Program introduced synthetic pyrethroid (SP) and alpha-cypermethrin (5% WP) in indoor residual spray (IRS) in 2015 [7].

Both DDT and pyrethroids target voltage-gated sodium channels; hence, our primary concern is that the prolonged use of a single class of insecticides may have induced selection pressure for resistance development in sand flies [8]. Currently, there is limited information about the susceptibility of *Ph. argentipes* to synthetic pyrethroids on the Indian subcontinent [9, 10]. Target-site insensitivity (TSI) and metabolic detoxification are well documented mechanisms for pyrethroid resistance [11]. The common target of synthetic pyrethroids and organochlorines is the *Vgsc* (voltage-gated sodium channel) gene on sand fly nerve cells. *Vgsc* are transmembrane proteins that facilitate the movement of sodium ions, and alterations in membrane potential regulate their activation and deactivation. Proper operation of these channels is crucial for the regular transmission of nerve signals. However, when insecticides bind to the channels, it disrupts the process and leads to paralysis and finally death [12, 13]. Multiple mutations in *Vgsc* genes, which confer target site insensitivity, have been demonstrated to be the underlying cause of pyrethroid resistance [14, 15]. The prolonged use of pyrethroids may lead to the emergence of insects that have reduced sensitivity to these insecticides at their target sites, a condition referred to as knockdown resistance (*kdr*). Most identified mutations linked to *kdr* involve a substitution of leucine with serine and phenylalanine at position 1014 of domain II segment 6 (DIIS6) of the *Vgsc* gene [16] and frequent mutations have been observed at L1014H, and less commonly L1014C and L1014W [17, 18]. There are multiple findings on the resistance of vector mosquito to insecticides and the genetic variations in the *Vgsc* gene [11, 19, 20]. However, there are few such reports available on sand flies specifically for *Ph. argentipes* [18, 21, 22].

Organophosphates (OPs) comprise a class of insecticides used for sand fly control, which act through the inhibition of acetylcholinesterase (AChE) in the central nervous system. Single nucleotide variants (SNV) in acetylcholinesterase-1 (*ace-1*) produce an altered, insensitive AChE mechanism of organophosphate- and carbamate-resistance. Sand fly biochemical studies have revealed possible reduced AChE susceptibility in *Ph. argentipes* field populations from Sri Lanka [23, 24]. The presence of known *ace-1* mutations (e.g., G119S) confer target-site insensitivity to OPs and carbamates in mosquitoes and other insects [25]. This is yet to be examined in *Ph. argentipes* sand flies.

IRS is one of the major tools used for VL elimination. Metabolic and target site insensitivity is the most studied and increasingly important for resistance surveillance because it allows for the early detection of the resistance mechanism potentially displayed phenotypically. It is concerning that so little is known regarding the mechanisms of insecticide resistance in *Phlebotomus* sand fly populations. Such knowledge is essential for public health officials to make informed, effective decisions about proper insecticide use to better control sand flies.

Insecticide resistance is a heritable, genetic trait and it is possible that field-caught flies will develop resistance over multiple, successive generations of exposure to sublethal doses of pyrethroids. The purpose of this study was to investigate the accumulation of insecticide resistance in *Ph. argentipes* captured in IRS and non-IRS villages in Muzaffarpur, Bihar via measurement of metabolic detoxification and target-site insensitivity in *Vgsc* and *ace-1* genes.

## Methods

### Study areas

**Fig. 1:**
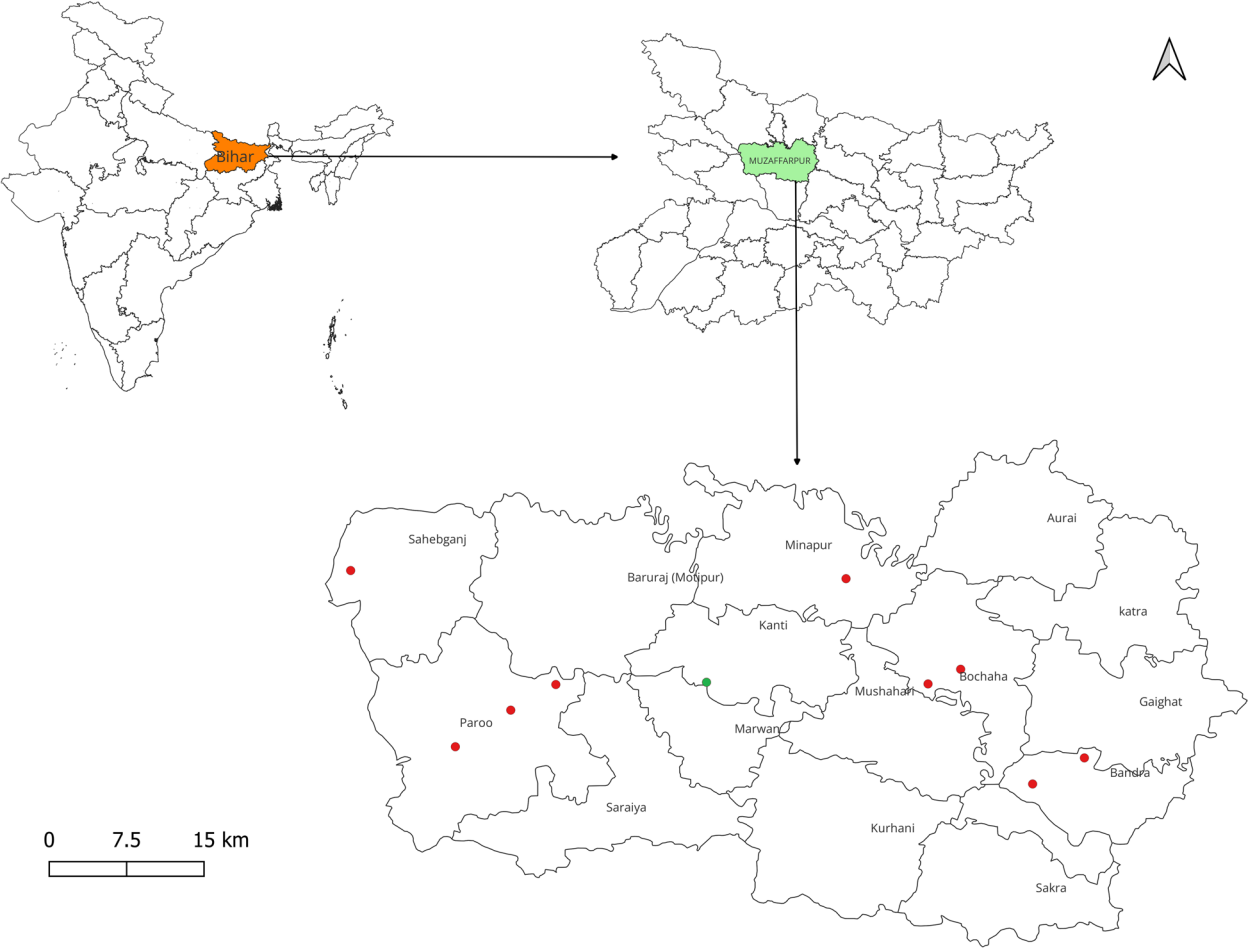
Geographical map of Muzaffarpur district showing the locations of the IRS and non-IRS villages included in the study. Red dots represented selected IRS villages while the green dot represented the non-IRS village. The map was produced using QGIS software (version 3.30.3), with open assess shapefile (https://onlinemaps.surveyofindia.gov.in)

The sand flies were captured from ten different villages known to have previously high incidence of VL in the Muzaffarpur district of Bihar. These villages are situated at coordinates 26°07′N, 85°24′E (Fig. 1). The villages were chosen on the basis of the yearly occurrence of VL cases and the continuous historical application of alpha-cypermethrin-based IRS. Nine villages were selected as IRS villages, where regular indoor residual spraying (IRS) was implemented from 2017 to 2022, due to the presence of patients with visceral leishmaniasis (VL). Villages reporting any case in a given year are sprayed with IRS for the following three consecutive years. All IRS villages have been sprayed with synthetic pyrethroid (SP) since 2016. One village, where no VL case was reported, was designated as a nonintervention village, and did not get any SP-IRS treatment (non-IRS village). IRS data have been collected from the Ministry of Health and the IRS team, and confirmed with the micro action plan for IRS of Muzaffarpur District. The IRS villages that were chosen for the study were Madhubani, Madhopur Hazari, Pandey, Simara, Hirapur, Hamidpur, Anandpur Kharuni, Noonfara, and Ramdash Majhauli, and Manifulkaha as the non-IRS village (Table 1).

**Table 1:** List of selected villages with high incidence of VL cases and historical application of IRS

Sr no.	Village	VL cases reported					PKDL cases reported					IRS with synthetic pyrethroid performed in
		2017	2018	2019	2020	2021	2017	2018	2019	2020	2021	
1	Manifulkaha*											
2	Ramdas Majhauli		1									2017, 2018, 2019, 2020, 2021
3	Madhubani	2	5	3	2					1		2017, 2018, 2019, 2020, 2021, 2022
4	Anandpur Kharuni	4	23	8	5	3	2		1	1	5	2017, 2018, 2019, 2020, 2021, 2022
5	Pandey	1	16	9	2	3		1	3		3	2017, 2018, 2019, 2020, 2021, 2022
6	Hirapur	4	6	1	3	2	3	1				2017, 2018, 2019, 2020, 2021, 2022
7	Madhopur Hazari	17	32	13	2	1		1	1	3	6	2017, 2018, 2019, 2020, 2021, 2022
8	Hamidpur	7	2	2	3	1						2017, 2018, 2019, 2020, 2021, 2022
9	Noonfara	3	2	2	5	1					1	2017, 2018, 2019, 2020, 2021, 2022
10	Simara	2	3	2	4	3		1				2017, 2018, 2019, 2020, 2021, 2022

^*^Non-IRS villages

### Sand fly collection from villages

Sand flies were collected on a monthly basis from March to November 2022 with CDC light traps installed in human dwellings, cattle sheds, and mixed dwellings (shared by both humans and cattle in the evening and operated overnight [26]). Early the following morning the traps and collection bags were taken to the Kala-azar Medical Research Centre (KAMRC), where the captured sand flies were removed and identified using morphological keys [27]. *Phlebotomus argentipes* sand flies were then stored at −20 °C awaiting further analysis.

### Biochemical assays

Specific activity levels of esterase, glutathione S-transferase, amounts of monooxygenases, and total protein were determined in ten wild-caught adult *Ph. argentipes* from each village [24, 28]. Each fly was individually homogenized in 200 µl of ice-cold distilled water. The homogenates were centrifuged for 30 s at 14,000 g. Aliquots of supernatant were analyzed in the following assay [24].

#### p-Nitrophenyl acetate (PNPA) esterase assay

Then, 200 ml of 1 mM pNPA working solution (100 mM pNPA in acetonitrile: 50 mM sodium phosphate buffer, pH 7.4, 1:100) were added to each 10 ml supernatant replicate. Enzyme rates were measured at 405 nm for 2 min at 22 °C. The pNPA activity per individual was reported as the rate of change in absorbance over time [24].

#### Glutathione S-transferase (GST) activity

For GST activity, two 10 µL replicates of each homogenate were placed in separate wells of a microtiter plate and mixed with the GSH/CDNB working solution (95 parts of 10.5 mM reduced glutathione in 100 mM phosphate buffer with five parts of 63 mM 1-chloro-2,4-dinitrobenzene, CDNB, in methanol). A reference well held distilled water. The plate was incubated at room temperature for 20 min, and absorbance was measured at 340 nm [24].

#### Cytochrome p450 monooxygenases

Two 20 µL replicates of each homogenate were placed in separate wells of a microtiter plate and mixed with 0.625 mM potassium phosphate buffer. Subsequently, 6.3 mM tetramethyl benzidine (TMBZ) working solution (0.01 g TMBZ dissolved in 5 ml methanol and then in 15 ml of sodium acetate buffer pH 5.0) and 3% (v/v) H_2_O_2_ solution were added. The plate was incubated at room temperature for 2 h and absorbance was measured at 650 nm [24].

#### Total protein

All sand flies were analyzed for total protein to calculate specific activity; 10 µL of homogenates were mixed with 200 µL protein reagents (Thermo Scientific Pierce, BCA protein assay kit, Rockford, USA). The plate was incubated at 37^0^C for 30 min, and absorbance was measured at 562 nm. Protein content in mg/ml was calculated using the standard curve of the known concentration of bovine serum albumin [24].

### DNA extraction and polymerase chain reaction

Genomic DNA was isolated from individual *Ph. argentipes* sand flies using a Gentra Puregene Tissue DNA Extraction Kit (Qiagen; Hilden, Germany) following the manufacturer’s instructions. A total of 10–25 *Ph. argentipes* were used per village; 166 *Ph. argentipes* used for Vgsc and 150 *Ph. argentipes* used for *ace-1* sequencing analysis. DNA was extracted using 30 μl of Milli-Q water and then stored at −20 °C for further processing. The study evaluated target-site insensitivity in the voltage-gated sodium channel and acetylcholinesterase (AChE) gene. The *Vgsc* primers (Vssc8F: 5′-AATGTGGGATTGCATGCTGG-3′, Vssc1bR: 5′-CGTATCATTGTCTGCAGTTGGT-3′) described by Gomes et al.[22] were used to amplify a genomic DNA fragment from *Vgsc* domain II, segment 6, which includes codons 1011, 1014, and 1016. The *ace-1* primers (F12:5ʹ-CAACGGATAAGGGGAAGG-3ʹ, R8:5ʹ-AAACCTGTGATCGTACAC-3ʹ) described by Denlinger et al. [29] were used to amplify a genomic DNA fragment from the *ace-1* gene, which includes the 119th position codon. All polymerase chain reaction (PCR) products were visually analyzed using gel electrophoresis with 1.5% TAE gels and purified using the QIAquick PCR Purification Kit (Qiagen; Hilden, Germany).

### Sanger sequencing

Samples were sent to Eurofins Genomics, Bangalore for automated Sanger sequencing using the same primers. The sequences were analyzed using Bio Edit Sequence Alignment Editor version 7.0.9.0 software [30]. The sequences were matched with the reference sequence for *Musca domestica* (accession no. U38813.1) at 1011th, 1014th, and 1016th codon position for *Vgsc* gene and 119th position for *ace-1* gene.

### Data analysis

An individual sample was classified as non-*kdr* and *kdr* genotypes. Those samples with either two wild-type leucine or leu/ser or leu/phe heterozygotes at 1014th position are considered non-*kdr* genotypes, while ser/ser, ser/phe or phe/phe are considered *kdr* genotypes. The *ace-1* gene, wild-type genotype has glycine amino acid at 119th position. Chi-squared test was used to assess the differences between allelic and genotypic frequencies at the *Vgsc* codon per village. Mann–Whitney *U* test was used to analyzed the difference in enzymatic activity. This nonparametric test was selected due to small sample size, independence of samples, and non-normal distribution of enzyme activity.

## Results

### Emerging metabolic resistance in the IRS village

**Fig. 2:**
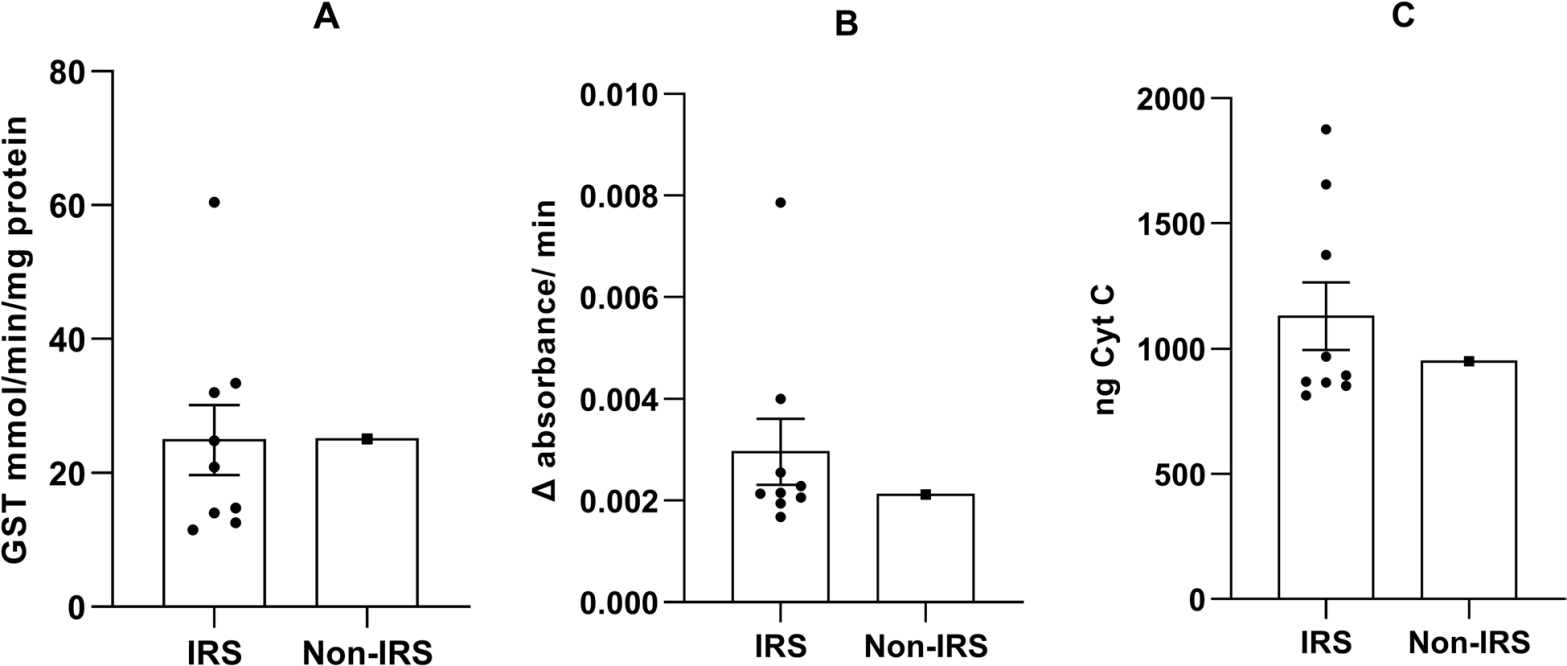
Comparison of detoxification enzyme activities in *Ph. argentipes* from IRS and non-IRS villages. **A** GST activity, **B** PNPA activity, and **C** monooxygenase activity. Bars represent the mean enzyme activity across IRS villages, and individual points indicate the mean value of pool sand fly samples for each village. Mean GST, PNPA, and MFO values were 24.94, 0.00296, and 1129.98 in IRS villages, respectively; while in non-IRS villages they were 25.08, 0.00212, and 950.15

Biochemical analysis of *Ph. argentipes* collected from nine IRS villages and one non-IRS village demonstrated varying levels of detoxification enzyme activities. The activity of glutathione S-transferase (GST) varied from 11.49 ± 1.54 to 60.40 ± 36.43 mMol/min/mg protein in IRS villages. The highest activity was found in Simara (60.40 ± 36.43), followed by Ramdash Majhauli (33.40 ± 31.33) (Table 2, Fig. 2). Manifulkaha, the non-IRS village, had moderate GST activity (25.08 ± 24.46). General esterase activity, assessed via p-nitrophenyl acetate (PNPA), showed the highest activity in Madhubani (0.00786 ± 0.005 Δ absorbance/min), higher than that in other villages (range 0.00168–0.0040), including the non-IRS village, Manifulkaha (0.00212 ± 0.00098) (Table 2, Fig. 2). This increased esterase activity in Madhubani may indicate a localized metabolic response that could facilitate insecticide detoxification.

**Table 2:** *Phlebotomus argentipes* mean enzyme activity (± standard deviation) for the glutathione S-transferase (GST), p-nitrophenyl acetate (PNPA), and mixed functional oxidases (MFO)

IRS status	Villages	GST mMol/min/mg protein	PNPA (Δ absorbance/min)	Monooxygenase (ng Cyt C)
IRS	Anandpur Kharuni	20.89 (17.36)	0.00215 (0.0002)	852.13 (207.43)
	Hamidpur	11.49 (1.54)	0.0040 (0.00035)	1875.51 (186.94)
	Hirapur	14.74 (7.43)	0.00214 (0.00022)	868.64 (56.25)
	Madhubani	12.54 (2.31)	0.00786 (0.005)	1656.12 (105.2)
	Noonfara	14.06 (7.35)	0.00195 (0.00015)	813.62 (107.82)
	Ramdash Majhauli	33.40 (31.33)	0.00229 (0.00047)	1374.8 (934.98)
	Simara	60.4 (36.43)	0.00255 (0.0005)	969.74 (108.8)
	Pandey	24.86 (17.92)	0.00206 (0.00015)	893.39 (84.57)
	Madhopur Hazari	32.05 (21.1)	0.00168 (0.0028)	865.88 (110.11)
Non-IRS	Manifulkaha	25.08 (24.46)	0.00212 (0.00098)	950.15 (148.52)

The activity of monooxygenase (cytochrome P450), measured in ng of cytochrome C equivalents, was significantly higher in the IRS villages of Hamidpur (1875.51 ± 186.94) and Madhubani (1656.12 ± 105.2) compared to the non-IRS village of Manifulkaha (950.15 ± 148.52). The average P450 activity in IRS villages (mean ± SD: 1179.32 ± 371.5 ng Cyt C) was higher than that of the non- IRS village (Table 2, Fig. 2). However, due to inter-village variability and the limitation of a single non-IRS village, there was no statistically significant difference (*P* > 0.05, Mann–Whitney *U* test). Despite this, small effect size suggests a steady rise in enzyme levels in IRS villages, especially Simara, Hamidpur, and Madhubani, suggesting metabolic detoxification mechanism in *Ph. argentipes* due to prolonged exposure to alpha-cypermethrin.

### High frequencies of serine and phenylalanine at 1014th position in the *Vgsc* gene from IRS villages

**Fig. 3:**
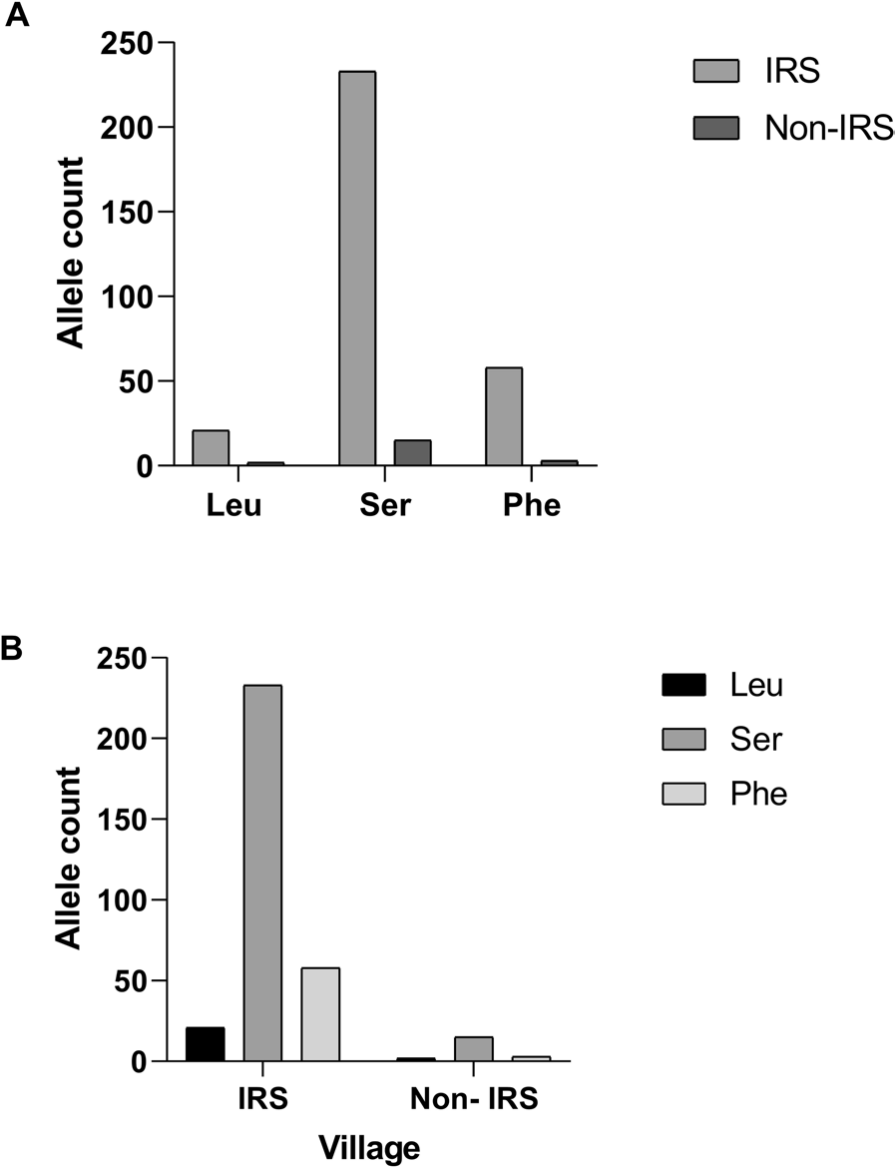
Allele distribution at position 1014 in *Phlebotomus argentipes* from IRS and non-IRS villages. **A** The bar chart shows alleles at codon 1014 across pool samples from nine IRS-treated villages (IRS) and one non-IRS village. **B** The bar represents raw allele counts. Statistical comparison of allele distributions between IRS villages and the non-IRS village was performed using chi-squared test (*P* = 0.8)

Sequence analysis of domains IIS6 of *Vgsc* in individual specimens of *Ph. argentipes* trapped from villages in the Muzaffarpur district revealed the presence of knockdown-resistant polymorphism at codon 1014. Mutations were identified due to the substitutions of leucine codon with serine (TTA → TCA, TTA → TCT, TTA → TCC), or leucine with phenylalanine (TTA → TTT, TTA → TTC). These mutations varied in frequency among villages (Table 3). The most frequent mutations were the L1014S codon (48.5%), followed by the wild-type L1014 codon (39.5%) (Fig. 3). For L1014S, the frequency was highest (19/20, 95%) in Hirapur and lowest in Ramdash Majhauli (7/17, 41%). A high frequency of L1014F was observed in Ramdash Majhauli and Noonfara (Supplementary Material 1: Table S1). High frequencies of phenylalanine at 1014 indicate a higher tolerance to synthetic pyrethroids. We did not observe any mutations at the 1011th and 1016th codons in *Vgsc*.

**Table 3:** Allele frequency at codon 1014 of *Vgsc*

IRS status	Village	*N*	Leu	Ser	Phe
IRS	Anandpur Kharuni	30	0.066	0.7	0.233
	Hamidpur	38	0.026	0.711	0.263
	Hirapur	40	0	0.975	0.025
	Madhubani	48	0.083	0.833	0.083
	Noonfara	32	0	0.687	0.313
	Ramdash Majhauli	34	0.059	0.588	0.352
	Simara	26	0.154	0.615	0.231
	Pandey	24	0.083	0.833	0.083
	Madhopur Hazari	40	0.15	0.75	0.1
Non-IRS	Manifulkaha	20	0.1	0.75	0.15

N is total number of alleles in each village, leu is wild-type allele, ser and phe are *kdr* allele. Mean allele frequencies in IRS villages were leu 0.067, ser 0.753, and phe 0.179, while in the non-IRS village they were leu 0.100, ser 0.750, and phe 0.150

### No mutation observed in 119th codon of *ace-1* gene

All *Phlebotomus* are genotyped to a glycine (GGA) at the 119th codon. No mutation at 119th codon from IRS villages and the non-IRS village indicates that sand flies may be sensitive to organophosphates and carbamates.

## Discussion

Indoor residual spraying plays a crucial role in making significant progress toward VL elimination. Knowledge of insecticide resistance mechanism in Phlebotomus is very limited. Our previous finding shows that insecticide exerts selection pressure and continuous, repeated exposure of synthetic pyrethroid led to develop the resistance in sand flies from Muzaffarpur, Bihar. In this present study, we investigated the underlying mechanisms of insecticide resistance in *Ph. argentipes* captured from IRS and non-IRS villages in Muzaffarpur, Bihar, India. Both metabolic detoxification and target-site insensitivity, particularly to synthetic pyrethroids, show the early evidence of resistance in *Phlebotomus*. In the post-elimination phase, monitoring the efficacy of IRS and the susceptibility status of *Ph. argentipes* is very much needed to sustain the elimination. Resistance to DDT has become well established and reported in *Ph. argentipes* in India [8], however, studies on resistance against pyrethroids in *Phlebotomus* sand flies are very limited in the Indian subcontinent. Like in mosquitoes, insecticide resistance in sand flies can be caused by target-site insensitivity (TSI) and metabolic detoxification (MD). Several reports have been published on the *kdr* mutation in *the Vgsc* gene in sand flies [15, 17–19], while very limited or no reports have been published on metabolic resistance and cuticular or behavioral resistance.

The biochemical assays provide strong indications of enhanced metabolic activity in the IRS villages compared with the non-IRS control village. Elevated levels of detoxification enzymes glutathione S-transferases (GSTs), esterases, and cytochrome P450 monooxygenases were observed in sand flies from Simara, Hamidpur, and Madhubani. Glutathione S-transferase (GST) activity was high in sand flies from Simara, suggesting a potential role of GSTs in conferring pyrethroid resistance through the detoxification of insecticide compounds. Elevated monooxygenase levels in flies from Hamidpur and Madhubani further support the hypothesis that oxidative metabolism may contribute to detoxifying insecticides and their degradation [31, 32]. Although some variation in enzyme levels among villages could be due to environmental or operational factors, the consistent pattern of increased enzyme activity in sand flies from IRS villages compared with those in non-IRS village strongly supports the notion that the increase in activity of metabolic enzymes might be due to selective pressure from repeated and continuous exposure to alpha-cypermethrin through IRS.

Target site insensitivity in the *Vgsc* gene is known as knockdown resistance (*kdr*) with mutations at 1011th, 1014th, and 1016th codons of the *Vgsc* gene reported, and potentially showing resistance against pyrethroid and DDT [33, 34]. In insects of agricultural and public health importance, the convergence of *kdr* at the 1014th codon demonstrates the biochemical significance of the leucine’s interaction with pyrethroids or DDT [35]. The native leucine is substituted for a phenylalanine, serine, histidine, cysteine, or tryptophan in *kdr* individuals [34]. In our study, we observed a high frequency of *kdr* mutations, L1014S and L1014F, similar to previous reported studies [18, 21]. Generally, one nucleotide substitution is involved in the *kdr* mutation, but more than one nucleotide mutation was found in our study. Sand flies from non-IRS villages have a high proportion of leu/ser heterozygotes, indicating previous exposure to DDT. A high proportion of phenylalanine homozygotes in sand flies from IRS villages indicates their tolerance to alpha-cypermethrin. Mutant allele frequencies were higher in flies from IRS villages, which may be due to selection pressure caused by the continuous exposure to synthetic pyrethroids.

The presence of *kdr* mutations in *Ph. argentipes* in Bihar, India, linking the presence of mutations to phenotypic data, was first described in 2017 [22]. While both L1014S and L1014F confer resistance to DDT, it is thought that L1014F confers stronger resistance to DDT than L1014S, and only two mutant alleles (phe/phe, phe/ser and ser/ser) are thought to produce a resistant phenotype. Phenylalanine frequencies in our study are comparatively higher than the previous studies in Bihar [18]. The widespread presence of *kdr* alleles suggests that the *Ph. argentipes* populations in Muzaffarpur are under significant selective pressure, likely driven by long-term use of alpha-cypermethrin. Our findings of higher frequencies of the mutant genotype and the recent emergence of pyrethroid resistance in sand flies in Nepal and India indicate that alternative insecticides should be incorporated into an integrated resistance management strategy [9, 10].

Interestingly, despite clear evidence of resistance to pyrethroids, our screening of the *ace-1* gene revealed no mutations at the G119 codon, which is typically associated with resistance to organophosphates and carbamates. This suggests that the *Ph. argentipes* populations in these regions remain susceptible to organophosphate-based insecticides. The continued sensitivity to organophosphates presents a strategic alternative for managing resistance, such as rotation or combination strategies, to delay or reverse resistance development.

Analyses of sand flies from the non-IRS village, Manifulkaha, clearly show moderate levels of enzyme activity and lower frequencies of *kdr* alleles. Thus, supporting the hypothesis that prolonged and repeated exposure to pyrethroids via IRS is the primary driver of insecticide resistance in sand fly populations from IRS villages. However, the presence of *kdr* alleles in sand flies from the non-IRS village may indicate gene flow from neighboring treated populations or residual effects of past insecticide use.

Knowledge of amino acid variations can be useful for vector control officials when determining insecticide rotations. The presence of *kdr* in sand flies could present a threat to the current IRS program used for VL control in endemic villages in India. Thus, regular susceptibility testing and efficacy of IRS are needed to control the sand fly vector and help sustain VL elimination. Use of different insecticides or a combination of insecticides should be explored for insecticide-resistance management and potential integration of vector control activities for VL and other vector-borne diseases. In conclusion, this study presents compelling evidence that *Ph. argentipes* sand flies in Bihar are developing both metabolic and genetic resistance mechanisms against synthetic pyrethroids due to prolonged exposure to insecticides. Future studies should expand resistance monitoring across a broader geographical range and investigate the fitness costs associated with resistance mutations to guide evidence-based insecticide policy in India.

The main limitations of our study are small sand fly populations selected to evaluate from each village. Villages were selected on the basis of insecticide phenotypic display and a history of spraying. We acknowledge that larger sample sizes would provide greater statistical power for population-level inference.

## Conclusions

Our study highlighted the presence of elevated metabolic enzymes and *kdr* mutations in the *Ph. argentipes* population exposed to alpha-cypermethrin-based IRS in Muzaffarpur, Bihar. This finding highlighted that repeated and continuous exposure to pyrethroids is driving the development of resistance in the sand fly population. The absence of *ace-1* mutations suggests that sand flies have not developed resistance to organophosphates. Our study emphasizes the urgent need for continuous monitoring of insecticide resistance in sand flies and the adoption of integrated vector management to sustain the ongoing efforts in eliminating visceral leishmaniasis.

## Supplementary Information

Supplementary Material 1. Table S1 Genotype frequencies at 1014 in IRS and non-IRS village

## Data Availability

Data supporting the main conclusions of this study are included in the manuscript.

## Abbreviation

*kdr*: Knockdown resistance
TSI: Target site insensitivity
DDT: Dichlorodiphenyltrichloroethane
OPs: Organophosphates
OCs: Organochlorine
IRS: Indoor residual spray
Vgsc: Voltage-gated sodium ion channel
AChE: Acetyl choline esterase
ISC: Indian subcontinent

## References

[1] Gedda MR, Madhukar P, Shukla A, Mudavath SL, Srivastava ON, Singh OP, et al. Nanodiagnostics in leishmaniasis: a new frontiers for early elimination. WIREs Nanomed Nanobiotechnol. 2021;13:e1675. 10.1002/WNAN.1675. 10.1002/wnan.1675 PMC7897294 33142369

[2] Sundar S, Rai M. Laboratory diagnosis of visceral leishmaniasis. Clin Vaccine Immunol. 2002;9:951. 10.1128/CDLI.9.5.951-958.2002. 10.1128/CDLI.9.5.951-958.2002 PMC120052 12204943

[3] Sundar S. The story of elimination of visceral leishmaniasis (kala-azar) in India—Challenges towards sustainment. PLoS Negl Trop Dis [Internet]. Public Library of Science; 2025 [cited 2026 Jan 11];19(8):e0013321. 10.1371/JOURNAL.PNTD.0013321. 40828749 10.1371/journal.pntd.0013321 PMC12364345

[4] Sundar S, Singh OP, Chakravarty J. Visceral leishmaniasis elimination targets in India, strategies for preventing resurgence. Expert Rev Anti-infect Ther. 2018;16:805–12. 10.1080/14787210.2018.1532790. 30289007 10.1080/14787210.2018.1532790 PMC6345646

[5] Kaul SM, Das RK, Shiv Raj SR, Saxena NBL, Narasimham M. Entomological monitoring of Kala-azar control in Bihar State, India: observations in Vaishali and Patna Districts. 1993

[6] Dinesh DS, Hassan F, Kumar V, Kesari S, Topno RK, Yadav RS. Insecticide susceptibility of *Phlebotomus argentipes* sandflies, vectors of visceral leishmaniasis in India. Trop Med Int Health. 2021;26:823–8. 10.1111/tmi.13576. 33733549 10.1111/tmi.13576

[7] Geneva. kala-azar elimination programme report of a who consultation of partners leish maniasis.

[8] Dhiman RC, Yadav RS. Insecticide resistance in phlebotomine sandflies in Southeast Asia with emphasis on the Indian subcontinent. Infect Dis Poverty. BioMed Central Ltd.; 2016; 5(1):106. 10.1186/s40249-016-0200-3. 27817749 10.1186/s40249-016-0200-3 PMC5098277

[9] Roy L, Uranw S, Cloots K, Smekens T, Kiran U, Pyakurel UR, et al. Susceptibility status of the wild-caught Phlebotomus argentipes (Diptera: Psychodidae: Phlebotominae), the sand fly vector of visceral leishmaniasis, to different insecticides in Nepal. PLoS Negl Trop Dis [Internet]. PLoS Negl Trop Dis; 2022 [cited 2024 Sep 19];16(7):e0010304. 10.1371/JOURNAL.PNTD.0010304. 35834563 10.1371/journal.pntd.0010304 PMC9321455

[10] Chaubey R, Shukla A, Kushwaha AK, Singh SK, Singh OP, Kumar R, et al. Monitoring alpha-cypermethrin susceptibility of Phlebotomus argentipes, the vector of visceral leishmaniasis in India, using the CDC bottle bioassay. Parasites and Vectors. BioMed Central Ltd. 2024;17:1–8. 10.1186/S13071-024-06579-W/FIGURES/3. 10.1186/s13071-024-06579-w PMC11622483 39639391

[11] Smith LB, Kasai S, Scott JG. Pyrethroid resistance in *Aedes aegypti* and *Aedes albopictus*: important mosquito vectors of human diseases. Pestic Biochem Physiol. 2016;133:1–12. 10.1016/J.PESTBP.2016.03.005. 27742355 10.1016/j.pestbp.2016.03.005

[12] O’Reilly AO, Khambay BPS, Williamson MS, Field LM, Wallace BA, Davies TGE. Modelling insecticide-binding sites in the voltage-gated sodium channel. Biochem J. 2006;396:255–63. 10.1042/BJ20051925. 16475981 10.1042/BJ20051925 PMC1462714

[13] Lund AE, Narahashi T. Kinetics of sodium channel modification as the basis for the variation in the nerve membrane effects of pyrethroids and DDT analogs. Pestic Biochem Physiol. 1983;20:203–16. 10.1016/0048-3575(83)90025-1.

[14] Rinkevich FD, Du Y, Dong K. Diversity and convergence of sodium channel mutations involved in resistance to pyrethroids. Pestic Biochem Physiol. 2013;106:93–100. 10.1016/J.PESTBP.2013.02.007. 24019556 10.1016/j.pestbp.2013.02.007 PMC3765034

[15] Davies TGE, Field LM, Usherwood PNR, Williamson MS. A comparative study of voltage-gated sodium channels in the Insecta: implications for pyrethroid resistance in Anopheline and other Neopteran species. Insect Mol Biol. 2007;16:361–75. 10.1111/J.1365-2583.2007.00733.X. 17433068 10.1111/j.1365-2583.2007.00733.x

[16] Burton MJ, Mellor IR, Duce IR, Davies TGE, Field LM, Williamson MS. Differential resistance of insect sodium channels with kdr mutations to deltamethrin, permethrin and DDT. Insect Biochem Mol Biol. 2011;41:723–32. 10.1016/J.IBMB.2011.05.004. 21640822 10.1016/j.ibmb.2011.05.004

[17] Wang ZM, Li CX, Xing D, Yu YH, Liu N, Xue RD, et al. Detection and widespread distribution of sodium channel alleles characteristic of insecticide resistance in *Culex pipiens* complex mosquitoes in China. Med Vet Entomol. 2012;26:228–32. 10.1111/J.1365-2915.2011.00985.X. 22070231 10.1111/j.1365-2915.2011.00985.x

[18] Kristan M, Hazelgrove C, Kumar K, Kumar A, Kumar V, Das P, et al. Knockdown resistance mutations in Phlebotomus argentipes sand flies in Bihar, India. Parasit Vectors. BioMed Central Ltd. 2024;17:1–8. 10.1186/S13071-024-06424-0/FIGURES/2. 10.1186/s13071-024-06424-0 PMC11311910 39123254

[19] Chatterjee M, Ballav S, Maji AK, Basu N, Sarkar BC, Saha P. Polymorphisms in voltage-gated sodium channel gene and susceptibility of Aedes albopictus to insecticides in three districts of northern West Bengal, India. PLoS Negl Trop Dis [Internet]. PLoS Negl Trop Dis; 2018 [cited 2024 Sep 20];12(1):e0006192. 10.1371/JOURNAL.PNTD.0006192. 29309419 10.1371/journal.pntd.0006192 PMC5774824

[20] Bharati M, Saha D. Multiple insecticide resistance mechanisms in primary dengue vector, Aedes aegypti (Linn.) from dengue endemic districts of sub-Himalayan West Bengal, India. PLoS One [Internet]. PLOS; 2018 [cited 2024 Sep 20];13(9):e0203207. 10.1371/JOURNAL.PONE.0203207. 30199543 10.1371/journal.pone.0203207 PMC6130861

[21] Reid E, Deb RM, Ali A, Singh RP, Mishra PK, Shepherd J, et al. Molecular surveillance of insecticide resistance in Phlebotomus argentipes targeted by indoor residual spraying for visceral leishmaniasis elimination in India. PLoS Negl Trop Dis [Internet]. PLoS Negl Trop Dis; 2023 [cited 2024 Sep 19]; 17(11):e0011734. 10.1371/JOURNAL.PNTD.0011734. 37939123 10.1371/journal.pntd.0011734 PMC10659200

[22] Gomes B, Purkait B, Deb RM, Rama A, Singh RP, Foster GM, et al. Knockdown resistance mutations predict DDT resistance and pyrethroid tolerance in the visceral leishmaniasis vector *Phlebotomus argentipes*. PLoS Negl Trop Dis. 2017;11:e0005504. 10.1371/JOURNAL.PNTD.0005504. 28414744 10.1371/journal.pntd.0005504 PMC5407848

[23] Pathirage DRK, Karunaratne SHPP, Senanayake SC, Karunaweera ND. Insecticide susceptibility of the sand fly leishmaniasis vector *Phlebotomus argentipes* in Sri Lanka. Parasit Vectors. 2020;13:1–12. 10.1186/S13071-020-04117-Y/TABLES/3. 32404115 10.1186/s13071-020-04117-y PMC7218544

[24] Surendran SN, Karunaratne SHPP, Adamsn Z, Hemingway J, Hawkes NJ. Molecular and biochemical characterization of a sand fly population from Sri Lanka: evidence for insecticide resistance due to altered esterases and insensitive acetylcholinesterase. Bull Entomol Res. 2005;95:371–80. 10.1079/BER2005368. 16048685 10.1079/ber2005368

[25] Essandoh J, Yawson AE, Weetman D. Acetylcholinesterase (Ace-1) target site mutation 119S is strongly diagnostic of carbamate and organophosphate resistance in *Anopheles gambiae* s.s. and *Anopheles coluzzii* across southern Ghana. Malar J. 2013;12:1–10. 10.1186/1475-2875-12-404/FIGURES/6. 24206629 10.1186/1475-2875-12-404 PMC3842805

[26] Tiwary P, Singh SK, Kushwaha AK, Rowton E, Sacks D, Singh OP, et al. Establishing, expanding, and certifying a closed colony of *Phlebotomus argentipes* (Diptera: Psychodidae) for xenodiagnostic studies at the Kala Azar Medical Research Center, Muzaffarpur, Bihar, India. J Med Entomol. 2017;54:1129–39. 10.1093/JME/TJX099. 28525618 10.1093/jme/tjx099 PMC5850120

[27] Ilango K. Phylogeny of the old world phlebotomine sandflies (Diptera: Psychodidae) with special reference to structural diversity of female spermathecae. Orient Insects. 2004;38:419–62. 10.1080/00305316.2004.10417409.

[28] Hemingway J. Insecticide resistance mechanisms (Field and laboratory manual).

[29] Denlinger D 2017 Understanding the mechanisms of insecticide resistance in *Phlebotomus papatasi* and *Lutzoymia longipalpis* Sand Flies (Diptera: Psychodidae: Phlebotominae). all graduate theses and dissertations, Spring 1920 to Summer 2023 10.26076/6454-295a

[30] Hall T 1999 Bioedit: a user-friendly biological sequence alignment editor and analysis program for windows 41 41 10.14601/phytopathol_mediterr-14998u1.29

[31] Vontas J, Katsavou E, Mavridis K. Cytochrome P450-based metabolic insecticide resistance in *Anopheles* and *Aedes* mosquito vectors: muddying the waters. Pestic Biochem Physiol. 2020. 10.1016/J.PESTBP.2020.104666. 32980073 10.1016/j.pestbp.2020.104666

[32] Fawaz EY, Zayed AB, Fahmy NT, Villinski JT, Hoel DF, Diclaro JW. Pyrethroid insecticide resistance mechanisms in the adult *Phlebotomus papatasi* (Diptera: Psychodidae). J Med Entomol. 2016;53:620–8. 10.1093/JME/TJV256. 26810731 10.1093/jme/tjv256

[33] Davies TGE, Field LM, Usherwood PNR, Williamson MS. DDT, pyrethrins, pyrethroids and insect sodium channels. IUBMB Life. 2007;59:151–62. 10.1080/15216540701352042. 17487686 10.1080/15216540701352042

[34] Dong K, Du Y, Rinkevich F, Nomura Y, Xu P, Wang L, et al. Molecular biology of insect sodium channels and pyrethroid resistance. Insect Biochem Mol Biol. 2014;50:1–17. 10.1016/J.IBMB.2014.03.012. 24704279 10.1016/j.ibmb.2014.03.012 PMC4484874

[35] Martinez-Torres D, Chandre F, Williamson MS, Darriet F, Bergé JB, Devonshire AL, et al. Molecular characterization of pyrethroid knockdown resistance (kdr) in the major malaria vector *Anopheles gambiae* s.s. Insect Mol Biol. 1998;7:179–84. 10.1046/J.1365-2583.1998.72062.X. 9535162 10.1046/j.1365-2583.1998.72062.x

