# Supplementary material for: Presence of localized elevated metabolic enzymes and kdr mutations in the voltage-gated sodium channel gene indicate early evidence of resistance of Phlebotomus argentipes to alpha-cypermethrin in Bihar, India
Source: Parasit Vectors. 2026 Mar 31;19:206. doi: 10.1186/s13071-026-07339-8 (PMC13159183; doi:10.1186/s13071-026-07339-8)
Supplement: Supplementary file 1 — Supplementary Material 1. Table S1 Genotype frequencies at 1014 in IRS and non-IRS village [file 13071_2026_7339_MOESM1_ESM.docx]

| IRS Status | Village | N | Leu/* | | | Total | Ser/ Ser | Ser/ Phe | Phe/ Phe |
| --- | --- | --- | --- | --- | --- | --- | --- | --- | --- |
|  |  |  | **Leu/**  **Leu** | **Leu/ Ser** | **Leu/ Phe** |  |  |  |  |
| IRS | Anandpur Kharuni | 15 | 0 | 2 | 0 | 2  (13.3) | 9  (60) | 1  (6) | 3  (20) |
|  | Hamidpur | 19 | 0 | 1 | 0 | 1  (5.2) | 13  (68.4) | 0 | 5  (26.3) |
|  | Hirapur | 20 | 0 | 0 | 0 | 0 | 19  (95) | 1  (5) | 0 |
|  | Madhubani | 24 | 0 | 3 | 1 | 4  (16.6) | 17  (70.8) | 3  (12.5) | 0 |
|  | Noonfara | 16 | 0 | 0 | 0 | 0 | 9  (56.2) | 4  (25) | 3  (18.7) |
|  | Ramdash Majhauli | 17 | 0 | 2 | 0 | 2  (11.7) | 7  (41.2) | 4  (23.5) | 4  (23.5) |
|  | Simara | 13 | 1 | 2 | 0 | 3  (23) | 7  (53.8) | 0 | 3  (23) |
|  | Pandey | 12 | 1 | 0 | 0 | 1  (8.33) | 10  (83.3) | 0 | 1  (8.33) |
|  | Madhopur Hazari | 20 | 0 | 5 | 1 | 6  (30) | 11  (55) | 3  (15) | 0 |
| Non-IRS | Manifulkaha | 10 | 1 | 0 | 0 | 1  (10) | 7  (70) | 1  (10) | 1  (10) |

**Table S1:** Genotype frequencies at codon 1014^th^ in IRS and non-IRS villages.

N= Number of Sand flies. Values in brackets are relative frequencies (percentages). Leu/* Showing the number of genotypes having at least one wild-type leucine; Ser/ Ser: Number of homozygotes for Serine. Ser/ Phe; Number of heterozygotes with serine and phenylalanine. Phe/ Phe; Number of homozygotes for Phe.
